# Healthcare resource utilisation and costs associated with AL amyloidosis: a retrospective matched cohort study

**DOI:** 10.1038/s41598-024-65654-5

**Published:** 2024-07-23

**Authors:** Shih-Pei Shen, Hsin-An Hou, Kuan-Chih Huang, Choo Hua Goh, Hong Qiu, Lee Anne Rothwell, Kwang-Wei Wu, Hitesh Chandwani, Yanfang Liu, Chao-Hsiun Tang

**Affiliations:** 1 School of Health Care Administration, College of Management, Taipei Medical University, 11F, Biomedical Technology Building, No.301, Yuantong Rd., Zhonghe Dist., New Taipei City, 235 Taiwan; 2 Division of Hematology, Department of Internal Medicine, National Taiwan University Hospital, Zhongzheng District, Taipei City, 100 Taiwan; 3 Global Epidemiology, Office of the Chief Medical Officer, Janssen Research & Development, LLC., Singapore, Singapore; 4 Global Epidemiology, Office of the Chief Medical Officer, Janssen Research & Development, LLC., Taipei, Taiwan; 5 Global Epidemiology, Office of the Chief Medical Officer, Janssen Research & Development, LLC., NJ, USA; 6 Janssen Medical Affairs Asia Pacific, North Ryde, Australia; 7 Janssen Medical Affairs Asia Pacific, Singapore, Singapore; 8 Janssen Market Access Asia Pacific, Singapore, Singapore; 9 Global Real-World Evidence, GCDS, GCSO, Janssen Research & Development LLC, Raritan, USA

**Keywords:** Haematological diseases, Health care economics

## Abstract

We conducted a retrospective population-based, matched cohort study using the National Health Insurance Research Database to estimate healthcare resource utilisation (HRU) and costs in patients with newly diagnosed AL amyloidosis in Taiwan. Cases were matched 10:1 by age, sex, and area of residence to patients without AL amyloidosis (comparators) randomly selected from the database during the same time period. Annual all-cause HRU and costs for 3 years were quantified. AL amyloidosis-attributable costs were obtained by subtracting all-cause HRU costs incurred by comparators from cases. The mean age of all patients was 60.78 years and 59.07% were male. Co-morbidities were more frequent in cases than comparators. By 6 months after diagnosis, 12.1% of cases had died versus 0.9% of comparators. In the first year, cases had 103% more outpatient visits, 177% more emergency room visits, were hospitalised 4-times more frequently, and spent 5.5-times more days in hospital than comparators, and total healthcare costs were > sixfold higher. Costs incurred during the first year after diagnosis accounted for 55% of the 3-year cumulative cost. High HRU costs associated with delayed diagnosis and end-organ damage indicate a need for earlier diagnosis and more effective treatments for AL amyloidosis.

## Introduction

Amyloidosis is a chronic, incurable disease caused by the accumulation of misfolded proteins that disrupt cellular activities and impair organ functioning. More than 30 proteins are known to form amyloid deposits, of which the most common are light chains (AL amyloidosis), caused by the production of excessive monoclonal immunoglobulin light chains by abnormal plasma cells^1^. The early symptoms of AL amyloidosis are frequently insidious and non-specific, leading to delays in diagnosis of up to several years^2,3^. As a result, many patients have advanced disease with heart, kidney and/or liver involvement at diagnosis, which correlates poorly with prognosis^4,5^.

Because of diagnostic delay or missed diagnosis, precise estimates about the incidence and prevalence of AL amyloidosis are difficult to make. The incidence has been estimated as 10 per million in Western countries and approximately 8.4 per million in Taiwan^6,7^. Treatment is based on chemotherapy directed against clonal plasma cells using combinations of steroids, cyclophosphamide, and novel agents including bortezomib, daratumumab, thalidomide, lenalidomide, pomalidomide. Autologous stem cell transplant (ACST) can induce hematologic remission and prolong survival^8^, however fewer than 25% of patients are transplant eligible due to underlying cardiac disease^3^. Treatment selection is determined by the extent of organ involvement, the presence of co-morbidities, and the presence or absence of the t(11;14) translocation which is associated with lower response rates to bortezomib therapy^3,9^.

Survival can be a matter of months in patients who present with advanced disease and extensive cardiac involvement, whereas patients diagnosed at an early stage can survive for more than 10 years^10^. The burden of disease for patients with AL amyloidosis is substantial—the heart is involved in 50–70% of patients, usually presenting as heart failure^11^. Approximately one-third of patients with renal involvement will eventually require dialysis, and a proportion of patients require organ transplant in order to tolerate chemotherapy^11^. A systematic review of the literature published in 2017 found that quality of life is significantly affected in patients with AL amyloidosis^11^.

Information about the baseline healthcare-related costs of AL amyloidosis is needed to inform health technology assessments for new treatments. Studies using healthcare claims databases in the United States (US) have reported that the total all-cause healthcare cost in the first year after a diagnosis of AL amyloidosis was US $122,180^12^. The cost of healthcare in the first year in patients with relapsed/refractory AL amyloidosis was US $139,143^13^. Outside of the US there has been little published about the cost of healthcare in patients diagnosed with AL amyloidosis^11^, and the burden of AL amyloidosis on healthcare systems in Asia is not known.

To bridge the gap in knowledge about the real-world economic burden of AL amyloidosis we conducted a population-based study using a national health insurance claims database to examine the healthcare resource utilisation (HRU) and costs associated with AL amyloidosis in Taiwan. AL amyloidosis-attributable HRU and costs were assessed by comparing outcomes in patients with and without AL amyloidosis.

## Methods

### Data source

This study analysed the National Health Insurance Research Database (NHIRD) provided by National Health Insurance (NHI) Administration and maintained by the Data Science Centre of the Ministry of Health and Welfare in Taiwan. The NHI programme is a compulsory national health insurance which covers 99.9% of the Taiwanese population, which was approximately 24 million people in 2020^14^. Once enrolled, members obtain easy access to a comprehensive benefits package that includes outpatient and inpatient services, dental care services, traditional medicine, prescription drugs, and laboratory and imaging examinations. Data captured by the NHI are released into the NHIRD, a large population-based claims database that contains information on all medical services provided by NHI-contracted hospitals, physicians, and pharmacies to residents throughout Taiwan. Demographic information as well as the dates and types of services received are captured in the database. All claims in the database are accompanied by diagnosis codes. International Classification of Diseases, Ninth Revision, Clinical Modification (ICD-9-CM) codes were available in the database up to December 31, 2015 and ICD-10-CM coding was introduced from January 1, 2016. All patients enrolled into the NHIRD have a unique ID that enables all healthcare episodes to be captured regardless of which clinic/hospital the patient attended. The survival status of members was verified by linking NHIRD to the Death Registry using scrambled identification numbers.

De-identified patient data were used for the analysis. The study was granted an exemption from ethical review by the Taipei Medical University-Joint Institutional Review Board, and an exemption from the need for patient consent. The study was conducted according to all applicable guidelines and regulations set by the Health and Welfare Data Science Center (HWDC).

### Study design and population

This is a retrospective population-based, matched cohort study to evaluate HRU and costs from the payer’s perspective. The study cohort included patients with newly diagnosed AL amyloidosis identified from the NHIRD using ICD-10 codes E85.4 (organ-limited amyloidosis), E85.8 (other amyloidosis), and E85.9 (amyloidosis, unspecified) between January 1, 2016 and December 31, 2018. The index date was defined as the first eligible occurrence of one of these codes. Eligible patients were those who fulfilled both of the following criteria: (1) had at least one inpatient claim(s) or at least two outpatient claims with primary or secondary diagnosis of AL amyloidosis, and with any pair of claims coming at least 30 days apart; and (2) underwent any biopsy procedure within 12 months prior to, or up to 6 months after the index date. Patients with any claim of amyloidosis with ICD-9 code 277.3 in 2015 in the NHIRD were excluded. Patients were also excluded if their initial diagnosis switched to another amyloidosis code (ICD 10: E85.0, E85.1, E85.2, E83.3) or if they had a record of non-AL amyloidosis (ICD-9: 277.3, ICD-10: E85.1) in the Catastrophic Illness database. Registration in the Catastrophic Illness database is considered confirmed because it requires meeting strict diagnostic evaluation criteria including biopsy confirmation and evaluation by two experts.

A comparator cohort of patients without AL amyloidosis was identified by randomly selecting individuals who had no claim for a diagnosis of amyloidosis from 2008 to 2019 in the database. Patients with newly diagnosed amyloidosis were matched with comparators in a ratio of 1:10 based on age, sex, and residential area, categorised into six areas defined by the NHI; Taipei area, Northern area, Central area, Southern area, Eastern area, and Kaohsiung and Ping-Tung area. Comparators were assigned to a reference date, which was the index date of the corresponding matched case.

The baseline period was defined as 365 days prior to the index/reference date. Relevant comorbidities (i.e., those potentially associated with or caused by amyloidosis)^15–19^ were defined using at least two outpatient or one inpatient claim(s) using ICD-9 and ICD-10 codes (Supplementary Table 1) during the baseline period. Co-morbidities were categorised into six major categories (cardiac-related conditions, liver-related diseases, renal-related diseases, pulmonary diseases, neuropathy, and malignancy).

### Outcomes

All-cause HRU and costs were captured for both cohorts on a per-patient basis from the index/reference date up until three years after the index date, death, or end of data availability (December 31st, 2019), whichever occurred first. Outpatient, inpatient, and emergency department services were evaluated. Total costs were further broken down by medication costs and non-medication costs (including fees for physician consultations, diagnostic tests, laboratory examinations, injection procedure costs, surgery and inpatient stays). By subtracting the *all-cause* HRU and costs incurred by matched comparators from the all-cause HRU and costs incurred by patients with AL amyloidosis (i.e., incremental HRU and costs), an estimate of the HRU and costs attributable to AL amyloidosis was obtained.

Costs were not inflated to a base year because health insurance claims within the NHI are mainly based on fixed fee schedules, and thus were reported as documented when the claims were filed from 2016 to 2019. Costs are presented in New Taiwan dollars (NT$). In mid-2022, 100 NT$ converts to around 3.2 Euros and 3.5 USD.

### Statistical analysis

Mean, standard deviation (SD), median, and interquartile range (IQR) were used to describe continuous variables, and frequencies and percentages were used to describe categorical variables. T-tests and Chi-square tests were applied to test the differences between the matched patients with AL amyloidosis and comparators for continuous and categorical variables, respectively.

Raw mean annual all-cause HRU and costs for 3 years were reported for patients with AL amyloidosis and comparators. Weighted Kaplan–Meier sample average (KMSA) estimates of HRU and costs were used to account for loss of follow-up due to death or disenrollment during the 3-year follow-up. This was achieved by charting the survival curves of patients with AL amyloidosis and comparators in the 3-year follow-up period using the Kaplan–Meier method. HRU and costs data within 3 years after the index date were then partitioned into 12 observation quarters for both cohorts. To account for the potential over-representation of zeros and right-skewed distribution of HRU and cost data, the mean quarterly HRU and costs were assessed using a two-part model: a logistic regression to predict the probability of service use and a generalised linear model to predict the frequency of HRU and costs among those with positive frequency of service. Covariates entered into the models to predict HRU and costs included age, sex, residential area, and the presence of any co-morbidity under the six relevant disease categories (cardiac-related conditions, liver-related diseases, renal-related diseases, pulmonary diseases, neuropathy, and malignancy).

The regression-predicted mean quarterly HRU, and costs of those patients still alive at the beginning of the quarter were weighted by the KMSA estimator, i.e., the probability of survival in the given quarter was conditional on having survived the previous quarter. This accounted for patient attrition over the study. The weighted estimates were then summed over to obtain an estimate of the mean censored adjusted cumulated HRU and costs up to the 3rd year^20–23^. This means that the weighted estimates were summed up to get an overall average HRU estimate, taking into account censoring (i.e., incomplete data) in the study population. By using weighted estimates, the results are representative of the larger population and the censored data are properly accounted for. This approach helps to provide a more accurate estimate by using all available information of HRU in the study population.

Since the mortality rate was relatively stable during the second and third year, the mean estimates of quarterly HRU and costs were collapsed into a semi-annual analysis for the second year and an annual analysis for the third year (Supplementary Tables 2 and 3).

Finally, a non-parametric bootstrap re-sampling method^7,8^ was performed to calculate the KMSA cost estimates via recycled predictions using 1,000 bootstrap samples of patients with AL amyloidosis and comparators of equal size with replacement. Two-sided 95% confidence intervals (CIs) were constructed.

All statistical analyses were performed using SAS Version 9.4 (SAS Institute, Cary, NC, USA).

## Results

In the 2016–2018 data from the NHIRD, 645 patients with newly diagnosed AL amyloidosis were identified with 6450 matched comparators (Table 1). The mean age of patients with AL amyloidosis and comparators was 60.78 years and 59.07% were male. Significantly more patients with AL amyloidosis had relevant co-morbidities; 27.29% of patients had cardiac-related conditions in the 365-day baseline period before their AL amyloidosis diagnosis compared to 12.11% of comparators, 23.41% had a malignancy compared to 4.43% of comparators and 21.71% had renal-related diseases compared to 3.80% of comparators. Eleven patients underwent autologous stem cell transplantation, all within the first 18 months after diagnosis.

**Table 1:** Demographic and clinical characteristics of patients with AL amyloidosis and comparators.

	Patients with AL amyloidosis (N = 645)		Comparators (N = 6450)		*P*
	n	%	N	%	
Gender					1.0000
Male	381	59.07	3810	59.07	
Female	264	40.93	2640	40.93	
Age at index date, years					1.0000
Mean ± SD	60.78 ± 14.16		60.78 ± 14.15		
Median (Q1, Q3)	61.05	(52.38, 70.4)	61.05	(52.38, 70.4)	
< 29	19	2.95	190	2.95	
30–39	29	4.50	290	4.50	
40–49	80	12.40	800	12.40	
50–59	172	26.67	1720	26.67	
60–79	177	27.44	1770	27.44	
70–79	112	17.36	1120	17.36	
> 80	56	8.68	560	8.68	
Residential areas					1.0000
Taipei area	169	26.20	1690	26.20	
North area	172	26.67	1720	26.67	
Central area	141	21.86	1410	21.86	
South area	73	11.32	730	11.32	
East area	11	1.71	110	1.71	
Kaohsiung & Ping-Tung area	79	12.25	790	12.25	
Comorbidities*					
Cardiac related conditions	176	27.29	781	12.11	< 0.0001
Malignancy	151	23.41	286	4.43	< 0.0001
Renal related diseases	140	21.71	245	3.80	< 0.0001
Pulmonary diseases	42	6.51	17	0.26	< 0.0001
Neuropathy	16	2.48	33	0.51	< 0.0001
Liver related diseases	15	2.33	6	0.09	< 0.0001

*SD* standard deviation, (*Q1*, *Q3*), interquartile range.

*Relevant co-morbidities were identified using ICD codes (Table S1) with at least two outpatient or one inpatient claim(s) during the baseline period.

### Mortality

**Figure 1:**
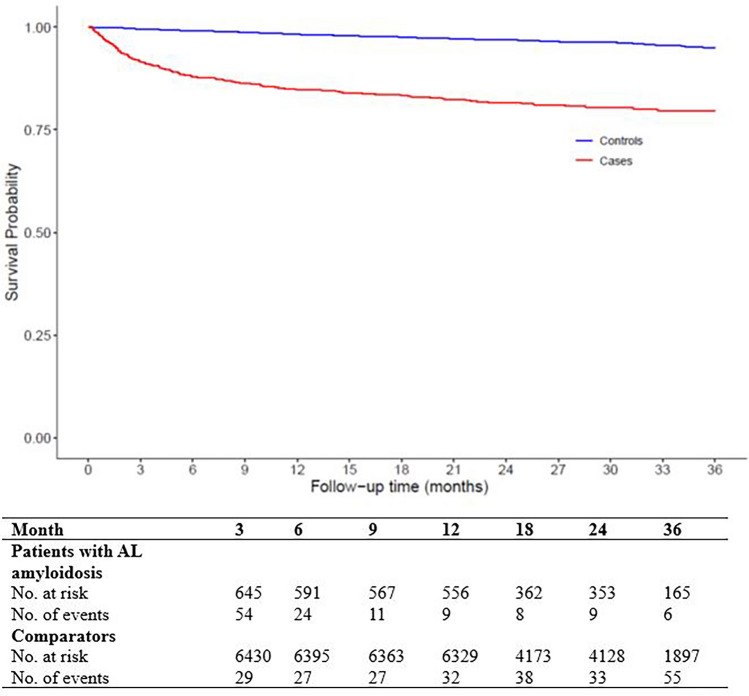
Kaplan–Meier estimates of survival in patients with newly diagnosed AL amyloidosis and comparators from the index date.

The Kaplan–Meier survival curve showed lower survival amongst patients with AL amyloidosis compared with comparators, and the majority of deaths occurred within the first 6 months after diagnosis (Fig. 1). Three months after the index date, 8.4% of patients with newly diagnosed amyloidosis died compared with 0.05% of comparators. By 6 months after the index date, 12.1% of patients had died, compared with 0.9% of comparators. By 36 months after the index date, 18.8% of patients had died compared with 3.7% of comparators.

### Healthcare utilisation

**Figure 2:**
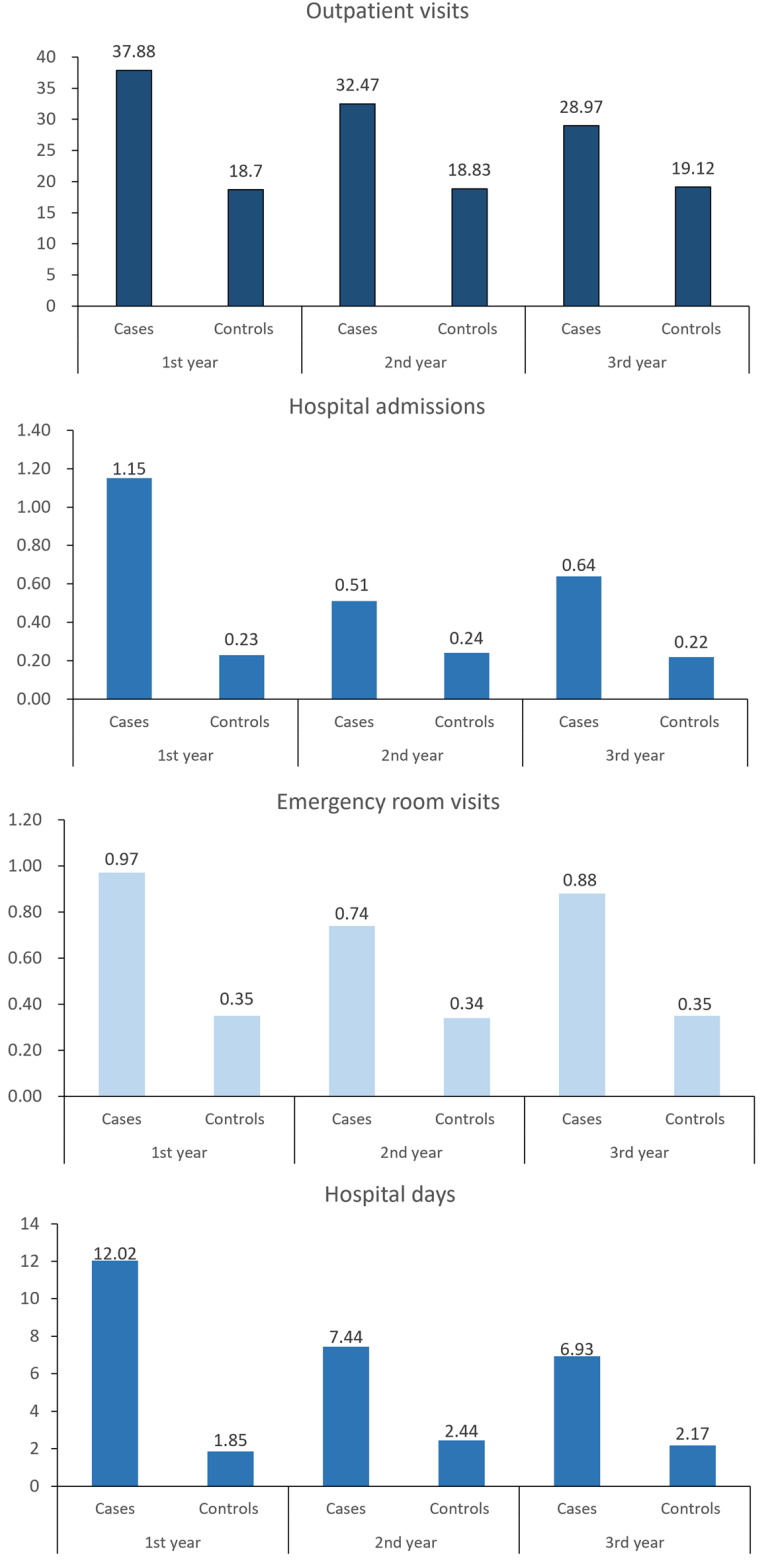
Crude means of annual outpatient visits, admissions, emergency room visits, and days spent in hospital, by each year after the index date for patients with newly diagnosed AL amyloidosis and comparators. Data are tabulated in Table S2.

Patients with newly diagnosed AL amyloidosis had levels of HRU that exceeded those of comparators. In the first year after the index date, patients with AL amyloidosis had a mean of 37.88 outpatient visits, 0.97 emergency room visits, and 1.15 hospitalisations, and spent 12.02 days in hospital (Fig. 2). Compared to healthcare utilisation by comparators, patients with newly diagnosed AL amyloidosis had 103% more outpatient visits, 177% more emergency room visits, were hospitalised 4-times more frequently, and spent 5.5-times more days in hospital. The difference between patients with AL amyloidosis and comparators decreased in year 2 and year 3, but HRU continued to remain substantially higher in patients than comparators.

HRU remained stable over the three follow-up years in comparators, whereas outpatient and emergency room visits, hospitalisations and days spent in hospital were all higher in the first year in patients with newly diagnosed AL amyloidosis and decreased thereafter. The biggest change from year 1 to year 2 was observed in the number of hospitalisations and days spent in hospital, which decreased by 56% and 38%, respectively in year 2 compared to year 1. Outpatient visits declined between year 2 and year 3, but hospitalisations and emergency room visits in patients increased in year 3 compared to year 2 (Fig. 2).

We explored HRU in patients and comparators for a period of 1 year prior to the index date. HRU in patients subsequently diagnosed with AL amyloidosis well exceeded that of comparators across all domains in the preceding year (Table S4).

### Costs of healthcare utilisation

**Figure 3:**
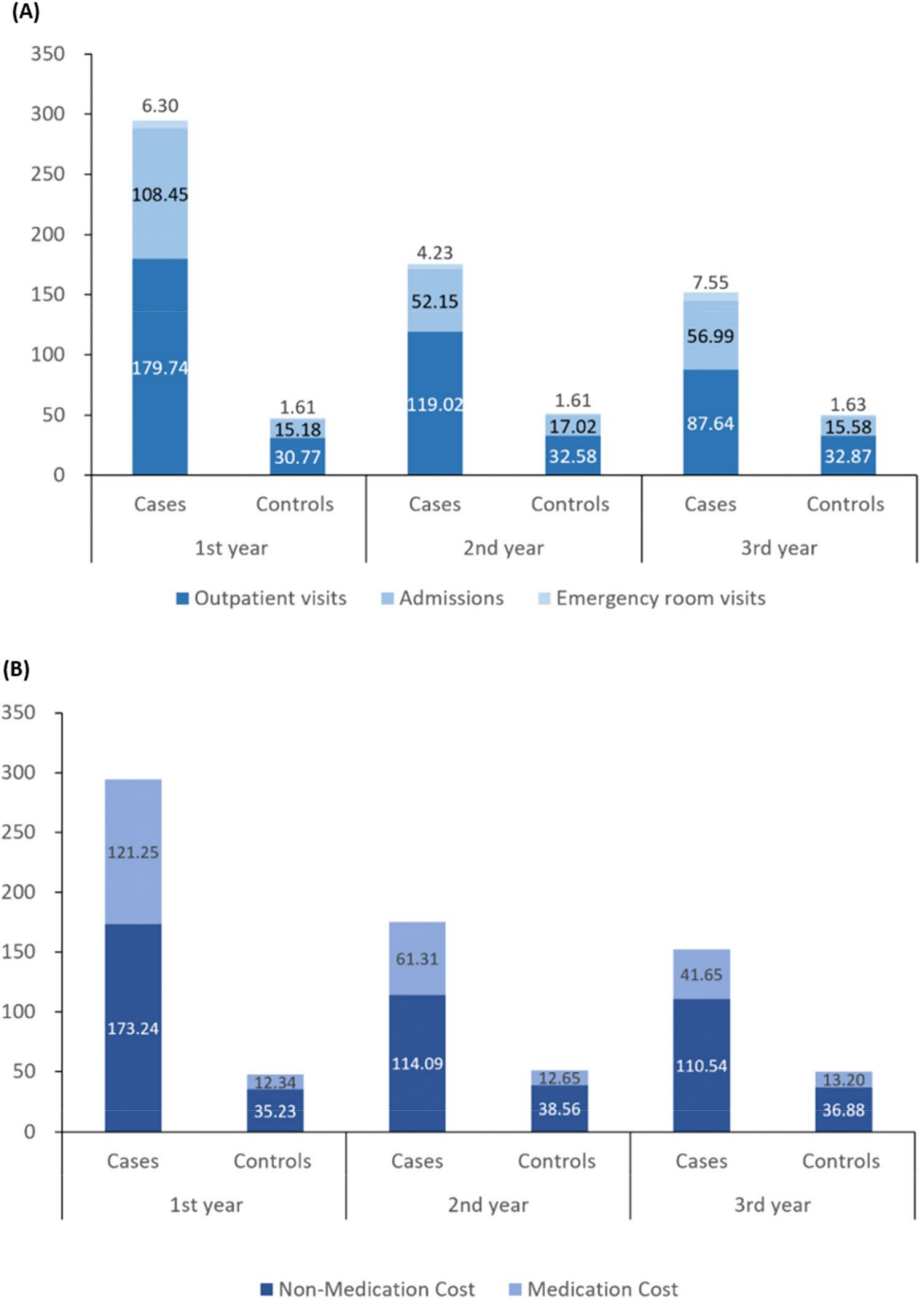
Crude mean annual total costs in NT$ per patient broken down by outpatient visits, admissions, and emergency room visits (**A**); and by medication costs and non-medication costs, by each year after the index date for patients with newly diagnosed AL amyloidosis and comparators (**B**). Data are tabulated in Table S3.

The mean total annual all-cause cost of HRU was NT$ 294,490 (USD 9,347) per patient in the first year after the index date versus NT$ 47,570 (USD 1,510) in comparators (Fig. 3A). In the first year, all-cause costs of outpatient visits accounted for 61% of the total cost (NT$ 179,740 [USD 5,705]), followed by hospitalisations (NT$ 108,450 [USD 3,442]) and emergency room visits (NT$ 6,300 [USD 200]). NT$ 121,250 (USD 3,848) was accrued in medication costs and NT$ 173,240 (USD 5498) in non-medication costs (Fig. 3B).

Costs accrued by hospitalisation and for medication were proportionally higher for patients with newly diagnosed AL amyloidosis than for comparators. Hospitalisation and medication costs accounted for 37% and 41%, respectively, of the all-cause total cost for year 1 in patients with AL amyloidosis.

Total mean total annual all-cause costs in patients decreased to NT$ 175,400 (USD 5,567) in year 2 and to NT$ 152,180 (USD 4,830) in year 3 yet remained at least threefold higher than costs accrued by comparators in each follow-up year.

**Figure 4:**
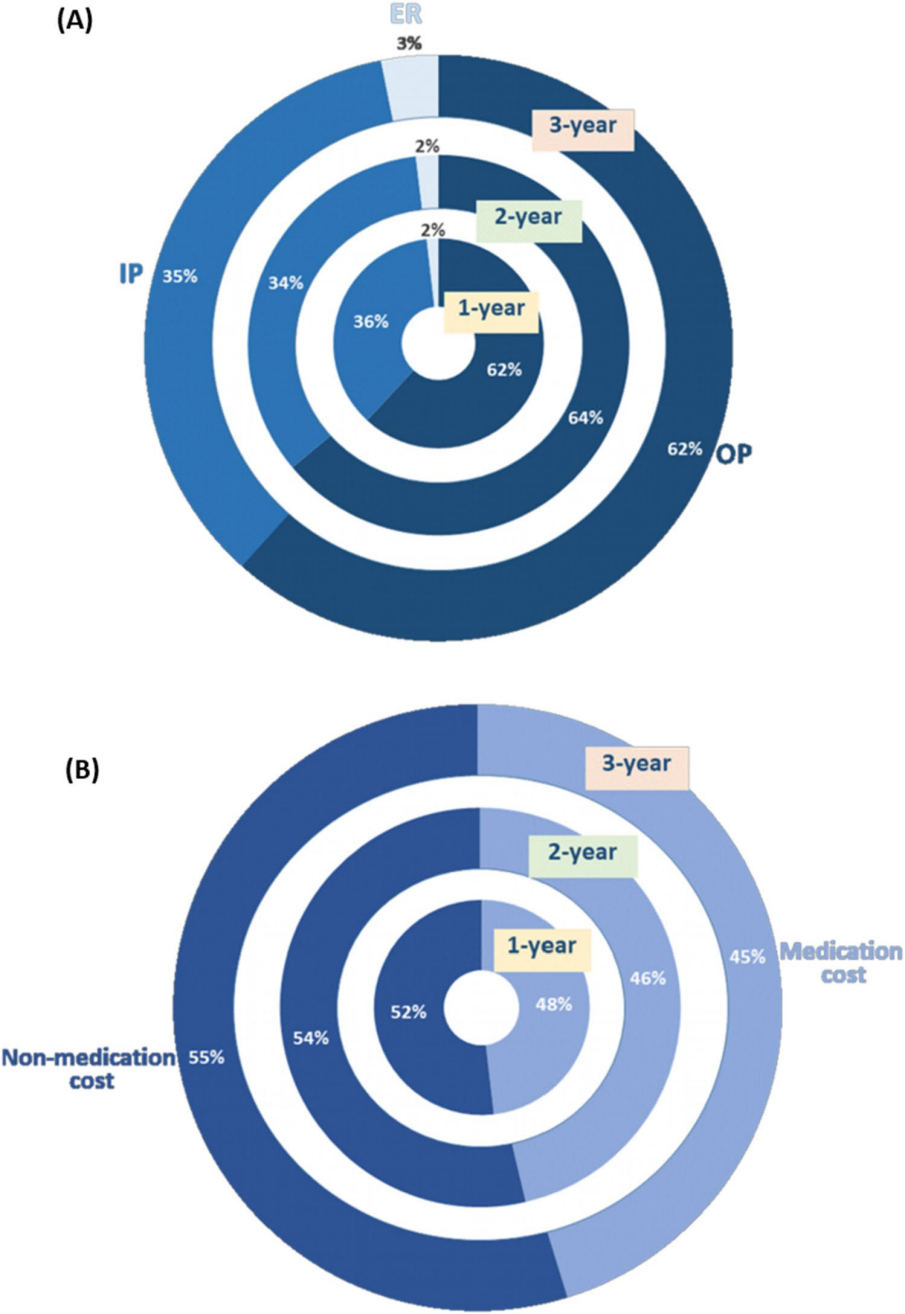
KMSA estimates of cumulative annual total costs of AL amyloidosis per patient broken down by (**A**) outpatient (OP), inpatient (IP), and emergency room (ER) care; and (**B**) medication costs and non-medication costs from the index date up to the third year.

Cumulative mean annual costs of HRU were significantly higher in patients with newly diagnosed AL amyloidosis than comparators over the entire 3-year follow-up period (*P* < 0.0001 for all comparisons) (Table 2). The mean total cost of HRU attributable to AL amyloidosis was NT$ 449,643 (SD 36,419) (USD 14,271, SD 1,156) after 3 years, made up of NT$ 259,560 (SD 20,379) (USD 8238, SD 646) for outpatient visits, NT$ 148,207 (SD 20,704) (USD 4704, SD 657) for inpatient episodes, and NT$ 13,242 (SD 4,575) (USD 420, SD 145) for emergency room visits (Table 2). Outpatient care contributed about 62%-64% and inpatient care 34%-36% to the 1-year, 2-year, and 3-year cumulated total costs (Fig. 4A). Costs incurred during the first year after diagnosis accounted for 55% of the 3-year cumulative cost. The total cost of all-cause healthcare after 3 years was fourfold higher in patients with AL amyloidosis compared to comparators.

**Table 2:** KMSA estimates of cumulative annual total costs per patient broken down by outpatient, inpatient, and emergency department from the index date up to the third year, for patients with newly diagnosed AL amyloidosis and comparators.

	Before bootstrapping		After bootstrapping			Comparators			Mean cost difference		
	Patients with AL amyloidosis	Comparators	Patients with AL amyloidosis								
	Mean	Mean	Mean	SD	95% CI	Mean	SD	95% CI	Mean	SD	*P* value*
(Unit: NT$)**											
Up to the end of 1st year											
Total Cost	304,457	48,700	296,006	22,910	294,584–297,427	48,275	1,975	48,153–48,398	247,730	16,260	< 0.0001
Outpatient visits	181,440	31,437	175,260	14,940	174,333–176,187	30,925	1,115	30,856–30,994	144,335	10,594	< 0.0001
Emergency room visits	5,738	1,588	5,821	646	5,781–5,861	1,593	98	1,587–1,599	4,228	462	< 0.0001
Hospitalisations	99,868	14,790	99,419	10,191	98,787–100,052	14,794	1,118	14,725–14,864	84,625	7,249	< 0.0001
Up to the end of 2nd year											
Total Cost	483,129	100,211	463,132	37,619	460,798–465,467	99,646	3,857	99,406–99,885	363,487	26,740	< 0.0001
Outpatient visits	296,971	63,726	283,079	23,581	281,616–284,542	63,096	2,257	62,956–63,236	219,983	16,751	< 0.0001
Emergency room visits	9,387	3,162	9,740	1,190	9,666–9,814	3,171	183	3,160–3,182	6,569	851	< 0.0001
Hospitalisations	148,582	31,285	149,125	18,448	147,981–150,270	31,344	2,111	31,213–3,1475	117,782	13,130	< 0.0001
Up to the end of 3rd year											
Total Cost	608,634	150,569	597,873	51,118	594,701–601,045	148,230	6,301	147,839–148,621	449,643	36,419	< 0.0001
Outpatient visits	365,937	96,865	354,666	28,491	352,898–356,434	95,106	4,341	94,837–95,376	259,560	20,379	< 0.0001
Emergency room visits	15,685	4,741	17,983	6,464	17,582–18,384	4,741	272	4,724–4,758	13,242	4,575	< 0.0001
Hospitalisations	192,301	45,680	194,150	29,116	192,343–195,957	45,943	3,089	45,751–46,135	148,207	20,704	< 0.0001

*CI* confidence interval; *KMSA* Kaplan–Meier sample average; *SD* standard deviation; *NT*$ New Taiwan dollars.

*t test; ** In mid-2022, 100 NT$ coverts to around 3.2 Euros and 3.5 USD.

Similarly, cumulative mean annual medication and non-medication costs were higher in patients than comparators over the entire 3-year follow-up period (*P* < 0.0001 for all comparisons) (Table 3). Medication costs consisted about 45%-48% of the 1-year, 2-year, and 3-year cumulated total costs (Fig. 4B). The mean total cost of medications attributable to AL amyloidosis was NT$ 212,606 (SD 27,497) (USD 6,748, SD 872) after 3 years, and the mean non-medication cost was NT$ 257,232 (SD 25,720) (USD 8,164, SD 816). 58% of the AL amyloidosis-attributable medication costs and 52% of the attributable non-medication costs over the 3-year follow-up period were incurred in the first year after diagnosis.

**Table 3:** KMSA estimates of cumulative annual total costs per patient broken down by medication costs and non-medication costs from the index date up to the third year, for patients with newly diagnosed AL amyloidosis and comparators.

	Before bootstrapping		After bootstrapping			Comparators			Mean cost difference		
	Patients with AL amyloidosis	Comparators	Patients with AL amyloidosis								
	Mean	Mean	Mean	SD	95% CI	Mean	SD	95% CI	Mean	SD	*P* value*
(Unit: NT$)**											
Up to the end of 1st year											
Total cost	304,457	48,700	296,006	22,910	294,584–297,427	48,275	1,975	48,153–48,398	247,730	16,260	< 0.0001
Medication cost	142,573	13,121	136,409	20,709	135,124–137,694	12,411	705	12,367–12,455	123,998	14,652	< 0.0001
Non-medication cost	171,102	35,635	169,076	13,382	168,246–169,907	35,717	1,575	35,619–35,814	133,360	9,528	< 0.0001
Up to the end of 2nd year											
Total cost	483,129	100,211	463,132	37,619	460,798–465,467	99,646	3,857	99,406–99,885	363,487	26,740	< 0.0001
Medication cost	214,654	25,540	198,158	27,861	196,429–199,887	24,905	1,400	24,819–24,992	173,253	19,725	< 0.0001
Non-medication cost	275,242	74,511	275,282	24,398	273,768–276,796	74,576	3,124	74,382–74,770	200,706	17,393	< 0.0001
Up to the end of 3rd year											
Total cost	608,634	150,569	597,873	51,118	594,701–601,045	148,230	6,301	147,839–148,621	449,643	36,419	< 0.0001
Medication cost	256,537	38,161	250,430	38,769	248,024–252,836	37,824	3,035	37,636–38,012	212,606	27,497	< 0.0001
Non-medication cost	363,521	111,441	367,475	36,071	365,237–369,714	110,243	4,683	109,952–110,533	257,232	25,720	< 0.0001

*CI* confidence interval; *KMSA* Kaplan–Meier sample average; *SD* standard deviation; *NT*$ New Taiwan dollars.

*t test; ** In mid-2022, 100 NT$ coverts to around 3.2 Euros and 3.5 USD.

Frequency of care and all health services, medication, and non-medication costs were substantially higher in the year prior to the index date in patients subsequently diagnosed with AL amyloidosis versus comparators (Table S5).

## Discussion

We used the NHIRD to identify patients in Taiwan with a new diagnosis of AL amyloidosis between 2016 and 2018 and assessed their mortality and HRU over the first three years after diagnosis. Patients with newly diagnosed AL amyloidosis had high levels of co-morbidity and high early mortality, with 12.1% of patients dying within 6 months after diagnosis. HRU and associated costs were substantially higher in patients than in comparators, and were highest in the first year after diagnosis, which likely reflects the burden of morbidity and early mortality amongst those patients who presented with advanced disease.

Delays in diagnosis and the late presentation of patients with severe end organ damage are well recognised challenges in the management of AL amyloidosis^2^. High rates of early mortality persist, despite improvements in overall survival that have followed the availability of more effective treatment options^2,24^. Higher costs incurred during the first year after diagnosis were observed in patients with AL amyloidosis in the US^25^; total all-cause healthcare costs decreased by 35% in the second year after diagnosis, which is similar to the 30% decrease in total all-cause healthcare costs observed in our study between year 1 and year 2. While total all-cause healthcare costs reduced by a further 13% in the third year after diagnosis in our study, total annual healthcare costs were still threefold higher than those in comparators.

We also observed higher HRU and associated costs in patients than in comparators in the year prior to diagnosis. Due to the insidious onset and chronicity of the disease, and because diagnosis may often be delayed, that HRU and cost estimates from the time of diagnosis do not reflect the entire burden of this disease on patients or the healthcare system.

There is no specific ICD code for AL amyloidosis and patients with AL amyloidosis may have multiple co-morbidities. Clinical data are not captured in claims databases and it is not possible to attribute causality to diseases that co-occur with AL amyloidosis. This means that disease-specific HRU and costs cannot be precisely identified in the NHIRD using claims with principal or secondary diagnosis codes of AL amyloidosis alone. By matching a cohort of patients without AL amyloidosis we were able to control for the occurrence of coincident co-morbidities in the regression model to determine the AL amyloid-attributable cost of healthcare over the first 3 years after diagnosis. We considered that relevant co-morbidities (i.e., those potentially associated with amyloidosis) in the AL amyloid population were unlikely to be present in the control population (without AL amyloidosis).

The AL amyloidosis-attributable cost was NT$ 247,730 (USD 7,862) in year 1, accumulating to NT$ 363,487 (11,537) by the second year, and NT$ 449,643 (USD 14,271) by the third year.

Potential limitations of our study are the absence of a specific diagnosis code in ICD-10 for AL amyloidosis, which is challenge common to all studies that use claims databases to evaluate this disease^12^. Such studies typically use a combination of disease and treatment codes to confirm the target diagnosis^12,13^. We were unable to incorporate treatment codes into our case definition because several therapies used for the treatment of AL amyloidosis, such as novel agents, were not re-imbursed by the NHI at the time of the study, and so were not captured in the NHIRD. Instead, we included a claim for any biopsy in the period 12 months before or 6 months after the index date as confirmatory evidence of an AL amyloidosis diagnosis. The validity of this approach has been reported previously^7^. Our study only considered direct healthcare costs and was not able to estimate the indirect costs associated with AL amyloidosis, or the effects on quality of life which is significantly impacted in AL amyloidosis^11^. Finally, we had no way of differentiating between localised and systemic amyloidosis in the claims database.

The main strength of our study is that the NHI is a mandatory health insurance for the whole population of Taiwan, and the NHIRD provides complete and longitudinal data collection from birth until death and provides a full picture of the treatment journey regardless of where the patient was treated. This eliminates issues related to data loss when patients are lost to follow-up.

Our study is the first to describe the healthcare burden associated with AL amyloidosis in an Asian country. We showed that patients with AL amyloidosis have a high burden of co-morbidities and suffer a high rate of early mortality in the first 6 months after diagnosis, suggesting that like other countries, disease awareness is low and diagnosis is often delayed in Taiwan. This pattern is also reflected in high rates of HRU and associated costs in the first year after diagnosis. The AL amyloidosis-attributable total cost of healthcare was NT$ 247,730 (USD 7,862) in the first year, accumulating to NT$ 449,643 (USD 14,271) by the third year. Costs incurred in outpatient care setting was the main cost driver, seconded by those incurred in the inpatient setting. Medication costs comprised almost half of the total medical costs in caring for patients with AL amyloidosis.

In conclusion, high HRU costs associated with delayed diagnosis and end-organ damage indicate a need for earlier diagnosis and more effective treatments for AL amyloidosis. More research is needed to understand the indirect costs associated with AL amyloidosis and the impact of this disease on the quality of life of patients in Asia.

### Supplementary Information

Supplementary Tables.

## Data Availability

The data underlying this study are from the NHIRD which has been transferred to the HWDC. The Taiwan government prohibits release of the NHI claims dataset to the public domain. Interested researchers can obtain the data through formal application to the HWDC, Department of Statistics, Ministry of Health and Welfare, Taiwan (http://dep.mohw.gov.tw/DOS/np-2497-113.html).
