# Supplementary material for: Healthcare resource utilisation and costs associated with AL amyloidosis: a retrospective matched cohort study
Source: Sci Rep. 2024 Jul 23;14:16915. doi: 10.1038/s41598-024-65654-5 (PMC11266401; doi:10.1038/s41598-024-65654-5)
Supplement: Supplementary file 1 — Supplementary Tables. [file 41598_2024_65654_MOESM1_ESM.pdf]

Healthcare resource utilisation and costs associated with AL amyloidosis: a retrospective matched cohort study

Shih-Pei Shen, Hsin-An Hou, Kuan-Chih Huang, Choo Hua Goh, Hong Qiu, Lee Anne Rothwell, Kwang-Wei Wu, Hitesh Chandwani, Yanfang Liu, Chao-Hsiun Tang

SUPPLEMENTARY INFORMATION

Supplementary Table 1. ICD-9 and ICD-10 codes of co-morbidities associated with AL amyloidosis

**Comorbidity**		**ICD-9 codes**	**ICD-10 codes**
Cardiac related conditions	Heart failure	428.x Heart failure<br>402.01 Malignant hypertensive heart disease with heart failure<br>402.11 Benign hypertensive heart disease with heart failure<br>402.91 Unspecified hypertensive heart disease with heart failure<br>404.01 Hypertensive heart and chronic kidney disease, malignant, with heart failure and with chronic kidney disease stage I through stage IV<br>404.03 Hypertensive heart and chronic kidney disease, malignant, with heart failure and with chronic kidney disease stage V or end stage renal disease<br>404.11 Hypertensive heart and chronic kidney disease, benign, with heart failure and with chronic kidney disease stage I through stage IV<br>404.13 Hypertensive heart and chronic kidney disease, benign, with heart failure and chronic kidney disease stage V or end stage renal disease<br>404.91 Hypertensive heart and chronic kidney disease, unspecified, with heart failure and with chronic kidney disease stage I through stage IV<br>404.93- Hypertensive heart and chronic kidney disease, unspecified, with heart failure and chronic kidney disease stage V or end stage renal disease	I50 Heart failure<br>I11 Hypertensive heart disease with (congestive) heart failure<br>I13.0 Hypertensive heart and renal disease with (congestive) heart failure<br>I13.2 Hypertensive heart and renal disease with both (congestive) heart failure and renal failure
	Heart block	426.0 Atrioventricular block, complete<br>426.1 Atrioventricular block other and unspecified<br>426.2 Left bundle branch hemiblock<br>426.3 Other left bundle branch block<br>426.4 Right bundle branch block<br>426.5 Bundle branch block other and unspecified<br>426.6 Other heart block<br>426.7 Anomalous atrioventricular excitation<br>426.8 Other specified conduction disorders<br>426.9 Conduction disorder, unspecified	I44 Atrioventricular and left bundle-branch block<br>I45 Other conduction disorders
	Arrhythmias	427.0 Paroxysmal supraventricular tachycardia<br>427.1 Paroxysmal ventricular tachycardia<br>427.2 Paroxysmal tachycardia, unspecified<br>427.4 Ventricular fibrillation and flutter<br>427.6 Premature beats<br>427.8 Other specified cardiac dysrhythmias<br>427.9 Cardiac dysrhythmia, unspecified	I47 Paroxysmal tachycardia<br>I49 Other cardiac arrhythmias
	Atrial Fibrillation	427.3 Atrial fibrillation and flutter	I48.0 Paroxysmal atrial fibrillation<br>I48.1 Persistent atrial fibrillation

Comorbidity		ICD-9 codes	ICD-10 codes
			I48.2 Chronic atrial fibrillation<br>I48.9 Atrial fibrillation and atrial flutter, unspecified
	Angina	413 Angina pectoris	I20 Angina pectoris
	Myocardial infarction	410 Acute myocardial infarction	I21 Acute myocardial infarction<br>I22 Subsequent myocardial infarction
	Pericardial effusion	423.9 Unspecified disease of pericardium	I31.3 Pericardial effusion (noninflammatory)
	Cardiomyopathy	425 Cardiomyopathy	I42 - Cardiomyopathy
Liver-related diseases	Hepatomegaly	789.1 Hepatomegaly	R16.0 Hepatomegaly, not elsewhere classified<br>R16.2 Hepatomegaly with splenomegaly, not elsewhere classified
	Liver ascites	789.59 Other ascites	R18 Ascites
	Acute liver failure	570 Acute and subacute necrosis of liver	K72.0 Acute and subacute hepatic failure
	Portal hypertension	572.3 Portal hypertension	K76.6 Portal hypertension
	Hepatorenal failure	572.4 Hepatorenal syndrome	K76.7 Hepatorenal syndrome
Renal-related diseases	Proteinuria	791.0 Proteinuria<br>593.6 Postural proteinuria	R80 Isolated proteinuria<br>N06 Isolated proteinuria with specified morphological lesion<br>N39.1 Persistent proteinuria, unspecified<br>N39.2 Orthostatic proteinuria, unspecified
	Nephrotic syndrome	581 Nephrotic syndrome	N04 – Nephrotic syndrome
	Chronic kidney disease	585.1 Chronic kidney disease, Stage I<br>585.2 Chronic kidney disease, Stage II<br>585.3 Chronic kidney disease, Stage III (moderate)<br>585.4 Chronic kidney disease, Stage IV (severe)<br>585.9 Chronic kidney disease, unspecified	N18.1 Chronic kidney disease, stage 1<br>N18.2 Chronic kidney disease, stage 2<br>N18.3 Chronic kidney disease, stage 3<br>N18.4 Chronic kidney disease, stage 4<br>N18.9 Chronic kidney disease, unspecified
	End-stage renal disease	585.5 Chronic kidney disease, Stage V<br>585.6 End stage renal disease	N18.5 Chronic kidney disease, stage 5<br>N18.6 End stage renal disease
Pulmonary diseases	Pleural effusion	511.1 Pleurisy with effusion, with mention of a bacterial cause other than tuberculosis<br>511.89 Other specified forms of effusion, except tuberculous convert<br>511.9 Unspecified pleural effusion	J90 Pleural effusion, not elsewhere classified<br>J91 Pleural effusion in conditions classified elsewhere

**Comorbidity**		**ICD-9 codes**	**ICD-10 codes**
Neuropathy	Carpel tunnel syndrome	354.0 Carpal tunnel syndrome	G56.0 Carpal tunnel syndrome
	Peripheral polyneuropathy	357.4 Polyneuropathy in other diseases classified elsewhere	G62.9 Polyneuropathy, unspecified<br>G63.3 Polyneuropathy in other endocrine and metabolic diseases
Malignancy	Solid tumour malignancy	140-149 Malignant Neoplasm Of Lip, Oral Cavity, And Pharynx	C00 – C14 Malignant neoplasms of lip, oral cavity and pharynx
		150-159 Malignant Neoplasm Of Digestive Organs And Peritoneum	C15 – C26 Malignant neoplasms of digestive organs
		160-165 Malignant Neoplasm Of Respiratory And Intrathoracic Organs	C30 – C39 Malignant neoplasms of respiratory and intrathoracic organs
		170 Malignant neoplasm of bone and articular cartilage	C40 – C41 Malignant neoplasms of bone and articular cartilage
		172 Malignant melanoma of skin<br>173 Other and unspecified malignant neoplasm of skin	C43 – C44Melanoma and other malignant neoplasms of skin
		171 Malignant neoplasm of connective and other soft tissue of head, face, and neck<br>176 Kaposi's sarcoma	C45 – C49 Malignant neoplasms of mesothelial and soft tissue
		174 Malignant neoplasm of female breast<br>175 Malignant neoplasm of male breast	C50 Malignant neoplasm of breast
		179 Malignant neoplasm of uterus, part unspecified<br>180 Malignant neoplasm of cervix uteri<br>181 Malignant neoplasm of placenta<br>182 Malignant neoplasm of body of uterus<br>183 Malignant neoplasm of ovary and other uterine adnexa<br>184 Malignant neoplasm of other and unspecified female genital organs	C51 – C58 Malignant neoplasms of female genital organs
		185 Malignant neoplasm of prostate<br>186 Malignant neoplasm of testis<br>187 Malignant neoplasm of penis and other male genital organs	C60 – C63 Malignant neoplasms of male genital organs
		188 Malignant neoplasm of bladder<br>189 Malignant neoplasm of kidney and other and unspecified urinary organs	C64 – C68 Malignant neoplasms of urinary tract
		190 Malignant neoplasm of eye<br>191 Malignant neoplasm of brain<br>192 Malignant neoplasm of other and unspecified parts of nervous system	C69-C72 Malignant neoplasms of eye, brain and other parts of central nervous system
		193 Malignant neoplasm of thyroid gland<br>194 Malignant neoplasm of other endocrine glands and related structures	C73-C75 Malignant neoplasms of thyroid and other endocrine glands
		195 Malignant neoplasm of other and ill-defined sites<br>199 Malignant neoplasm without specification of site	C76 Malignant neoplasm of other and ill-defined sites

Comorbidity		ICD-9 codes	ICD-10 codes
			C80 Malignant neoplasm, without specification of site
	Hematological malignancy	201 Hodgkin's disease	C81.x Hodgkin lymphoma
		202.0 Nodular lymphoma	C82.x Follicular lymphoma
		200.0 Reticulosarcoma<br>200.1 Lymphosarcoma<br>200.2 Burkitt's tumor or lymphoma<br>200.4 Mantle cell lymphoma<br>200.5 Primary central nervous system lymphoma<br>200.7 Large cell lymphoma<br>200.8 Other named variants of lymphosarcoma and reticulosarcoma	C83.x Non-follicular lymphoma
		200.6 Anaplastic large cell lymphoma<br>202.2 Sezary's disease<br>202.7 Peripheral t-cell lymphoma	C84.x Mature T/NK-cell lymphomas
		202.8 Other malignant lymphomas	C85.x Other and unspecified types of non-Hodgkin lymphoma<br>C86.x Other specified types of T/NK-cell lymphoma
		-	C88.0 – Waldenström macroglobulinaemia
		200.3 Marginal zone lymphoma<br>203.8 Other immunoproliferative neoplasms	Other malignant immunoproliferative diseases<br>C88.2 Other heavy chain disease<br>C88.3 Immunoproliferative small intestinal disease<br>C88.4 Extranodal marginal zone B-cell lymphoma of mucosa-associated lymphoid tissue (MALT-lyphoma)<br>C88.7 Other malignant immunoproliferative diseases<br>C88.9 Malignant immunoproliferative disease, unspecified
		203.0 Multiple myeloma	C90.0 Multiple myeloma
		203.1 Plasma cell leukemia	Other malignant plasma cell neoplasms<br>C90.1 Plasma cell leukaemia<br>C90.2 Extramedullary plasmacytoma<br>C90.3 Solitary plasmacytoma
		204 Lymphoid leukemia	C91.x Lymphoid leukaemia
		205 Myeloid leukemia	C92.x Myeloid leukaemia

Comorbidity		ICD-9 codes	ICD-10 codes
		206 Monocytic leukemia	C93.x Monocytic leukaemia
		207 Other specified leukemia	C94.x Other leukaemias of specified cell type
		208 Leukemia of unspecified cell type	C95.x Leukaemia of unspecified cell type
		202.3 Malignant histiocytosis<br>202.5 Letterer-siwe disease<br>202.6 Malignant mast cell tumors<br>202.9 Other and unspecified malignant neoplasms of lymphoid and histiocytic tissue	C96.x Other and unspecified malignant neoplasms of lymphoid, haematopoietic and related tissue
		-	D47.2 Monoclonal gammopathy of undetermined significance (MGUS)

Supplementary Table 2. Crude mean quarterly all-cause healthcare resource utilisation for patients with AL amyloidosis (comparators).

	Patients with AL amyloidosis					Comparators				
		Proportion of usage		Among users of care			Proportion of usage		Among users of care	
	N	n	%	Mean	SD	N	n	%	Mean	SD
**Frequency of care**										
**Outpatient visits**										
1st year 1st quarter	591	588	99.49	11.43	6.83	6401	5264	82.24	5.71	4.92
1st year 2nd quarter	567	547	96.47	9.39	6.62	6368	5240	82.29	5.60	4.88
1st year 3rd quarter	556	540	97.12	9.00	6.16	6336	5234	82.61	5.71	4.94
1st year 4th quarter	547	528	96.53	9.02	6.35	6297	5219	82.88	5.64	4.93
2nd year 1st & 2nd quarters	354	346	97.74	17.1	12.07	4132	3696	89.45	10.57	9.06
2nd year 3rd & 4th quarters	344	335	97.38	16.18	12.14	4094	3680	89.89	10.44	9.05
3rd year	159	159	100.00	28.97	17.72	1840	1750	95.11	20.1	16.90
**Emergency care visits**										
1st year 1st quarter	591	114	19.29	1.84	1.32	6401	397	6.20	1.37	0.96
1st year 2nd quarter	567	89	15.70	1.48	0.8	6368	401	6.30	1.32	1.09
1st year 3rd quarter	556	69	12.41	1.48	1.12	6336	434	6.85	1.39	1.34
1st year 4th quarter	547	70	12.80	1.53	0.96	6297	379	6.02	1.34	0.92
2nd year 1st & 2nd quarters	354	72	20.34	1.83	1.65	4132	491	11.88	1.45	0.99
2nd year 3rd & 4th quarters	344	69	20.06	1.86	1.23	4094	450	10.99	1.51	1.32
3rd year	159	59	37.11	2.37	1.80	1840	394	21.41	1.62	1.13
**Hospitalisations**										
1st year 1st quarter	591	175	29.61	1.67	0.97	6401	247	3.86	1.31	0.87
1st year 2nd quarter	567	102	17.99	1.47	0.69	6368	262	4.11	1.34	0.85
1st year 3rd quarter	556	75	13.49	1.43	0.74	6336	276	4.36	1.32	0.71
1st year 4th quarter	547	77	14.08	1.45	0.88	6297	266	4.22	1.30	0.73
2nd year 1st & 2nd quarters	354	54	15.25	1.56	0.86	4132	333	8.06	1.44	0.94
2nd year 3rd & 4th quarters	344	57	16.57	1.63	0.98	4094	314	7.67	1.5	1.19
3rd year	159	49	30.82	2.08	2.23	1840	243	13.21	1.67	1.25
**Hospital days**										
1st year 1st quarter	591	175	29.61	17.66	18.37	6401	247	3.86	9.67	11.82
1st year 2nd quarter	567	102	17.99	13.3	15.03	6368	262	4.11	10.83	16.07
1st year 3rd quarter	556	75	13.49	16.04	16.49	6336	276	4.36	11.83	16.42
1st year 4th quarter	547	77	14.08	15.94	19.05	6297	266	4.22	12.11	18.31

	Patients with AL amyloidosis					Comparators				
		Proportion of usage		Among users of care			Proportion of usage		Among users of care	
	N	n	%	Mean	SD	N	n	%	Mean	SD
2nd year 1st & 2nd quarters	354	54	15.25	20.98	34.73	4132	333	8.06	15.17	27.59
2nd year 3rd & 4th quarters	344	57	16.57	25.58	39.51	4094	314	7.67	15.86	28.49
3rd year	159	49	30.82	22.49	55.71	1840	243	13.21	16.44	40.26

Supplementary Table 3. Crude mean quarterly all-cause cost for patients with AL amyloidosis and comparators.

	Patients with AL amyloidosis					Comparators				
		Proportion of usage		Among users of care			Proportion of usage		Among users of care	
	N	n	%	Mean (NT$)	SD	N	n	%	Mean (NT$)	SD
**Cost of care (NT$)**										
**Total Cost**										
1st year 1st quarter	591	591	100.00	105,909.74	189,487.06	6401	5282	82.52	13,947.99	44,585.37
1st year 2nd quarter	567	549	96.83	74,470.45	146,313.75	6368	5258	82.57	14,141.29	56,833.02
1st year 3rd quarter	556	541	97.30	62,824.61	116,626.23	6336	5255	82.94	14,893.49	55,299.40
1st year 4th quarter	547	529	96.71	57,230.29	113,688.13	6297	5236	83.15	14,468.74	43,902.88
2nd year 1st & 2nd quarters	354	346	97.74	92,179.63	173,764.46	4132	3705	89.67	28,452.23	83,105.22
2nd year 3rd & 4th quarters	344	337	97.97	87,073.07	184,612.01	4094	3695	90.25	28,472.79	76,568.14
3rd year	159	159	100.00	152,183.01	294,070.51	1840	1753	95.27	52,568.89	140,610.18
**Broken down by types of service**										
**Outpatient visits**										
1st year 1st quarter	591	588	99.49	54,571.37	102,013.58	6401	5264	82.24	9,383.86	24,327.79
1st year 2nd quarter	567	547	96.47	49,803.76	98,168.35	6368	5240	82.29	9,085.87	22,230.45
1st year 3rd quarter	556	540	97.12	42,474.07	84,284.67	6336	5234	82.61	9,404.92	22,522.96
1st year 4th quarter	547	528	96.53	37,449.77	73,302.56	6297	5219	82.88	9,423.93	24,126.74
2nd year 1st & 2nd quarters	354	346	97.74	67,335.77	131,978.60	4132	3696	89.45	17,997.70	45,163.75
2nd year 3rd & 4th quarters	344	335	97.38	54,636.29	103,171.42	4094	3680	89.89	18,338.72	46,395.79
3rd year	159	159	100.00	87,643.23	139,821.31	1840	1750	95.11	34,561.80	93,553.33
**Emergency care visits**										
1st year 1st quarter	591	114	19.29	12,246.28	13,204.63	6401	397	6.20	5,838.60	7,172.09
1st year 2nd quarter	567	89	15.70	10,144.47	11,153.01	6368	401	6.30	5,712.25	8,110.74
1st year 3rd quarter	556	69	12.41	9,035.41	11,746.41	6336	434	6.85	7,102.52	11,367.11
1st year 4th quarter	547	70	12.80	9,602.91	11,407.92	6297	379	6.02	6,722.42	10,805.54
2nd year 1st & 2nd quarters	354	72	20.34	10,420.08	11,926.87	4132	491	11.88	6,879.48	8,680.32
2nd year 3rd & 4th quarters	344	69	20.06	10,508.16	13,992.68	4094	450	10.99	7,210.73	10,422.40
3rd year	159	59	37.11	20,352.03	54,746.82	1840	394	21.41	7,613.50	13,381.39
**Hospitalisations**										
1st year 1st quarter	591	175	29.61	166,334.94	232,475.15	6401	247	3.86	88,901.73	134,522.42
1st year 2nd quarter	567	102	17.99	124,889.80	168,601.77	6368	262	4.11	93,337.11	205,327.11

1st year 3rd quarter	556	75	13.49	139,048.98	125,585.03	6336	276	4.36	94,048.89	182,747.08
1st year 4th quarter	547	77	14.08	127,651.22	164,100.83	6297	266	4.22	90,327.31	116,592.01
2nd year 1st & 2nd quarters	354	54	15.25	145,291.28	187,193.32	4132	333	8.06	106,661.25	175,799.63
2nd year 3rd & 4th quarters	344	57	16.57	180,972.00	278,522.51	4094	314	7.67	109,794.98	140,803.83
3rd year	159	49	30.82	184,919.50	385,914.50	1840	243	13.21	117,985.17	207,507.12
**Broken down by medication and non-medication**										
**Medication Cost**										
1st year 1st quarter	591	586	99.15	40,316.52	102,998.13	6401	4504	70.36	4,367.80	18,281.24
1st year 2nd quarter	567	527	92.95	37,682.60	108,492.26	6368	4494	70.57	4,309.87	17,668.05
1st year 3rd quarter	556	520	93.53	27,012.81	77,043.32	6336	4496	70.96	4,381.54	17,652.03
1st year 4th quarter	547	508	92.87	22,598.38	66,312.98	6297	4492	71.34	4,369.29	18,039.16
2nd year 1st & 2nd quarters	354	338	95.48	36,819.31	108,068.09	4132	3281	79.40	8,089.84	34,902.60
2nd year 3rd & 4th quarters	344	322	93.60	27,943.00	83,492.84	4094	3248	79.34	7,842.52	30,669.59
3rd year	159	158	99.37	41,910.40	109,617.30	1840	1622	88.15	14,972.68	74,420.71
**Non-Medication Cost**										
1st year 1st quarter	591	591	100.00	65,934.31	125,669.11	6401	5282	82.52	10,223.54	36,209.11
1st year 2nd quarter	567	549	96.83	38,297.89	75,521.54	6368	5258	82.57	10,457.65	49,523.61
1st year 3rd quarter	556	541	97.30	36,860.35	71,896.31	6336	5255	82.94	11,144.80	46,469.71
1st year 4th quarter	547	529	96.71	35,529.02	81,466.78	6297	5236	83.15	10,720.30	35,216.95
2nd year 1st & 2nd quarters	354	346	97.74	56,211.63	116,285.28	4132	3705	89.67	21,288.19	65,833.02
2nd year 3rd & 4th quarters	344	337	97.97	60,373.82	139,036.78	4094	3695	90.25	21,579.01	64,169.58
3rd year	159	159	100.00	110,536.19	235,177.97	1840	1753	95.27	38,715.10	105,416.18

Supplementary Table 4. Healthcare utilization within one year before the index date.

Crude mean year all-cause healthcare resource utilization and cost for patients and comparators

	Patients with AL amyloidosis					Comparators				
		Proportion of usage		Among users of care			Proportion of usage		Among users of care	
	N	n	%	Mean	SD	N	n	%	Mean	SD
**Frequency of care**										
Outpatient visits	645	643	99.69	35.29	22.36	6450	6070	94.11	19.66	16.89
Emergency care visits	645	286	44.34	2.39	2.03	6450	1226	19.01	1.71	1.94
Hospitalisations	645	336	52.09	2.08	1.56	6450	729	11.30	1.55	1.27
Hospital days	645	336	52.09	18.47	22.38	6450	729	11.30	14.43	28.87
**Cost of care (NT$)**										
Total Cost	645	645	100.00	189758.83	286731.95	6450	6075	94.19	45125.12	111144.57
Broken down by types of service										
Outpatient visits	645	643	99.69	94995.71	168877.13	6450	6070	94.11	31153.79	74131.54
Emergency care visits	645	286	44.34	14578.34	19412.17	6450	1226	19.01	7267.12	13153.99
Hospitalisations	645	336	52.09	170067.87	242280.27	6450	729	11.30	104419.89	160150.08
Broken down by medication and non-medication										
Medication Cost	645	641	99.38	46966.3	138334.13	6450	5610	86.98	12057.73	40567.11
Non-Medication Cost	645	645	100.00	143083.8	219121.72	6450	6075	94.19	33990.33	91056.73

Supplementary Table 5. Healthcare resource utilization and cost within one year before the index date

	Patients with AL amyloidosis (n=645)	Comparators (n=6450)
	Mean	Mean
**Frequency of care**		
Outpatient visits	35.18	18.5
Emergency care visits	1.06	0.33
Hospitalisations	1.08	0.18
Hospital days	9.62	1.63
**Cost of care (NT$)**		
Total Cost	189758.83	42501.56
Broken down by types of service		
Outpatient visits	94701.15	29318.37
Emergency care visits	6464.19	1381.32
Hospitalisations	88593.49	11801.88
Broken down by medication and non-medication		
Medication Cost	46675.04	10487.42
Non-Medication Cost	143083.8	32014.14
